# Lifespan Mental Activity Predicts Diminished Rate of Hippocampal Atrophy

**DOI:** 10.1371/journal.pone.0002598

**Published:** 2008-07-09

**Authors:** Michael J. Valenzuela, Perminder Sachdev, Wei Wen, Xiaohua Chen, Henry Brodaty

**Affiliations:** 1 School of Psychiatry, University of New South Wales, Sydney, Australia; 2 The Neuropsychiatric Institute, Prince of Wales Hospital, Sydney, Australia; 3 Aged Care Psychiatry, Prince of Wales Hospital, Sydney, Australia; 4 Primary Dementia Collaborative Research Centre, University of New South Wales, Sydney, Australia; Indiana University, United States of America

## Abstract

**Objective:**

Epidemiological studies suggest that complex mental activity may reduce the risk for dementia, however an underlying mechanism remains unclear. Our objective was to determine whether individual differences in lifespan complex mental activity are linked to altered rates of hippocampal atrophy independent of global measures of neurodegeneration.

**Methods:**

Thirty seven healthy older individuals had their complex mental activity levels estimated using the Lifetime of Experiences Questionnaire (LEQ) and completed serial MRI investigations at baseline and three years follow-up. Hippocampal volume and semi-automatic quantitation of whole brain volume (WBV) and white matter hyperintensities (WMHs) were compared at both time points.

**Results:**

Higher LEQ scores were correlated with hippocampal volume independent of covariates at the three year follow-up stage (r = 0.43, p = 0.012). Moreover, those with higher LEQ scores experienced less hippocampal atrophy over the follow-up period (r = 0.41, p = 0.02). High LEQ individuals had less than half the hippocampal volume decline of low LEQ individuals in a multivariate analysis (F = 4.47, p = 0.042). No parallel changes were found in measures of WBV and WMHs.

**Conclusions:**

High level of complex mental activity across the lifespan was correlated with a reduced rate of hippocampal atrophy. This finding could not be explained by general differences in intracranial volume, larger hippocampi at baseline, presence of hypertensive disease, gender or low mood. Our results suggest that neuroprotection in medial temporal lobe may be one mechanism underlying the link between mental activity and lower rates of dementia observed in population-based studies. Additional studies are required to further explore this novel finding.

## Introduction

Epidemiological and clinical studies suggest that lifespan mental activity may be a significant modifiable protective factor in the development of dementia. A comprehensive systematic review found that individuals with a rich history of education, occupational complexity and cognitive lifestyle activities are at almost one-half the risk for developing dementia than those with less complex experiences [1]. Healthy ageing studies of cognition also supports this view [2], and a recent meta-analysis of longitudinal clinical trials suggests that cognitive training can slow the rate of mental decline in older individuals [3].

The underlying mechanism by which complex mental activity may affect development of dementia or cognitive decline are complex and are often referred to generically as *brain reserve* or *cognitive reserve* [4]. Potential mechanisms include disease modification as well as neuroprotective, neurotrophic, neuroregenerative and compensatory network responses to complex mental activity[5]. Evidence for a neuroprotective effect has come mainly from animal work investigating environmental enrichment, whereby mice given more opportunities for cognitive, social and physical activity are compared to those in standard housing. For example, in models of Huntington disease, enrichment delays disease onset, increases peristriatal volume and reverses associated neurotrophic abnormalities [6], [7]. Alzheimer's disease (AD) transgenic rodent studies have produced mixed results: three groups have reported between 30–50% reduction in amyloid burden [8]–[10], but others have replicated a behavioral advantage and found either increased β-amyloid burden [11], or no change [12]. Differences between how these groups operationally defined their specific enrichment intervention may partly account for these divergent findings.

Human research linking neuroprotection with mental activity is more limited. In the Nun Study, sisters with high levels of linguistic complexity in their early 20 s were almost 3-times *less* likely to have neurofibrillary tangles in their hippocampi at post mortem than those with low linguistic complexity [13]. Alternatively, others have preferred to argue this as evidence of subclinical impairment and disease up to 50 years prior to typical dementia onset [14].

The involvement of a specific neuroprotective mechanism in the medial temporal lobe related to complex mental activity is theoretically attractive given that neuropathology is believed to originate in this region [15], and atrophy of the hippocampus is a sensitive biomarker for early AD [16] [17]. Furthermore, a degree of gross morphological responsiveness to mental activity is not implausible since temporary increases in neocortical volume have been observed after a few weeks of behavioral training in younger adults [18].

Whether complex mental activity across the lifespan is related to hippocampal volume in late life, or the rate of hippocampal atrophy over time, has yet to be examined. Similarly, whether any putative neuroprotection is specific to the hippocampus or is also evident in more global measures of neurodegeneration is not known. Our aim was to therefore assess mental activity in a cohort of healthy older individuals at baseline and compare longitudinal hippocampal and whole brain volumetric change over three years with a view to further understand the link between complex mental activity and dementia risk.

## Results

### Characteristics of the Sample

**Table 1** shows the sociodemographic characteristics of the complete baseline sample, those with an initial MRI, the complete three year sample, and those that did or did not complete follow-up MRI. There were no systematic differences between these groups. Specifically, there were no significant age, education, NART-IQ, LEQ or MMSE differences between those who did or did not complete follow-up MRI at the 3 year follow-up stage.

**Table 1:** Cohort characteristics at baseline and follow-up.

	Baseline with LEQ	3 Year Follow Up	3 Year Follow Up With MRI ^1^	3 Year Follow Up Without MRI ^1^	p-value^1^
	N = 79	N = 70	N = 37	N = 33	
Age	70.9 (6.4)	70.3 (5.9)	70.3 (5.8)	71.9 (6.9)	0.3
Range:	58–93	58–85	58–83	60–93	
Gender (% Male)*	56.8	57.1	43.2	40.0	0.9
Education	12.0 (3.4)	12.1 (3.5)	12.04 (3.9)	12.0 (3.2)	0.9
Range:	7–25	7–25	7–25	8–19	
MMSE	28.7 (1.5)	28.8 (1.4)	28.8 (1.4)	28.7(1.7)	0.8
Range:	23–30	23–30	24–30	23–30	
NART-IQ	114.8 (8.1)	115.4 (7.5)	115.0 (7.3)	115.5 (8.9)	0.6
Range:	83–129	97–129	97–127	83–129	
% Hypertension*	35.8	34.3	29.5	42.9	0.3
LEQ Total	78.7 (24.2)	79.8 (23.8)	77.5 (21.4)	78.5 (27.1)	0.9
Range:	29.3–130.1	29.3–130.1	29.3–118.2	33.7–130.1	

27 follow-up subjects either refused or were not able to attend follow-up MRI or had scans with insufficient quality for quantitative analysis. There were no significant demographic or behavioural differences between those that did or did not undergo MRI at 3 years (p-value^1^). All p-values refer to t-test comparisons except for Chi-square analysis marked^*^.

### Complex Mental Activity and Brain Volumetry

**Figure 1:**
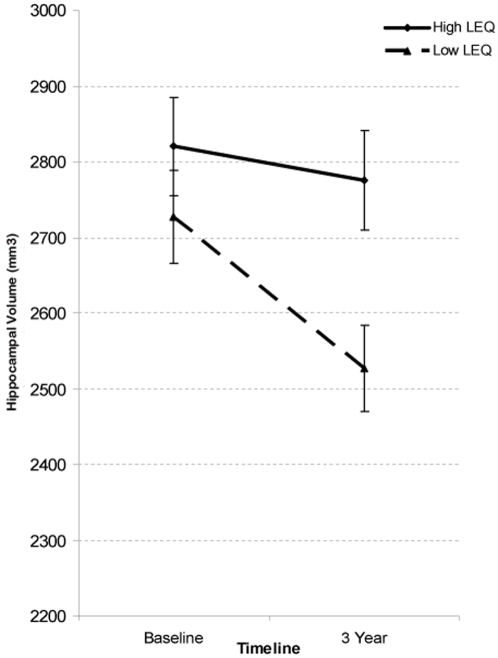
Average hippocampal volume (across right and left sides) in high (solid) and low (dashed) LEQ groups at baseline and 3 year follow-up. Error bars represent standard error of mean. *p-value after covariate control for age, gender, hypertension, baseline volume and total intracranial volume.

The relationship between LEQ and various neurodegenerative measures was examined. See **Table 2** for comparisons between the high and low LEQ groups. Hippocampal volume change in the two LEQ groups is displayed in **Figure 1**.

**Table 2:** Comparison of different neurodegenerative markers in high and low brain mental activity groups.

Neurodegenerative Marker	Low LEQ	High LEQ	Statistic
	N = 19	N = 18	
Total Hippocampal Volume _baseline_ (mm^3^)	2753.0 (333.6)	2849.6 (333.1)	F = 1.062, p = 0.31
Total Hippocampal Volume _3 Year_ (mm^3^)	2528.0 (270.0)	2747.6 (283.0)	F = 6.53, p = 0.016*
Total Hippocampal Volume _Change_ (mm^3^)	−225.0 (352.7)	−102.0 (312.5)	F = 5.25, p = 0.029*†
WBV _baseline_ (L)	1.08 (0.11)	1.08 (0.08)	F = 0.14, p = 0.68
WBV _3 Year_ (L)	1.10 (0.11)	1.12 (0.09)	F = 1.11, p = 0.30
WBV _Change_ (L)	0.01 (0.05)	0.04 (0.04)	F = 3.0, p = 0.096
Total WMH volume _baseline_ (mm^3^)	17870.2 (11059.8)	12594.8 (11381.5)	F = 0.10, p = 0.75
Total WMH volume _3 Year_ (mm^3^)	23261.1 (15054.8)	17605.2 (13685.3)	F = 0.02, p = 0.90
Total WMH volume _Change_ (mm^3^)	5390.8 (8084.7)	5010.4 (4387.3)	F = 0.10, p = 0.75

Variables were compared using the ANCOVA procedure whilst controlling for age, hypertension (HT), gender and multiple comparisons. All change analyses included the additional covariate of the respective baseline measure. ^†^ Addition of TICV covariate did not change results (F = 5.97, p = 0.02). High and low LEQ was by median-split. WBV: Whole Brain Volume, WMH: White matter hyperintensity.

**Figure 2:**
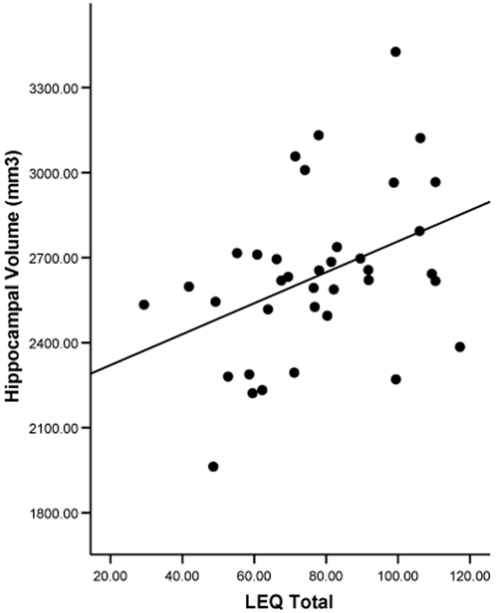
Scatterplot of total LEQ and hippocampal volume at three year follow-up. Zero-order correlation is shown in dashed line (r = 0.42, p = 0.006).

**Figure 3:**
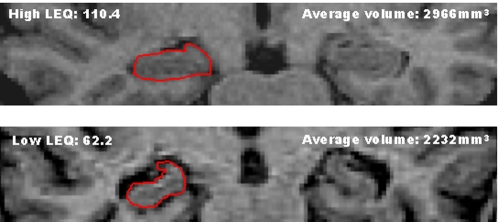
Examples of hippocampal volumes from a high and low LEQ individual at the 3 year follow-up stage. Note the relative increase in the volume of the inferior horn of the lateral ventricle. Average volumes refer to the mean across right and left sides. Right hippocampus tracing shown in red.

There were no significant partial correlation between LEQ and baseline hippocampal volume (r = 0.15, p = 0.40) after controlling for age, gender, hypertension and ICV. There was, however, a significant association between total LEQ and average hippocampal volume at the 3 year stage after control of age, gender, hypertension and ICV (r = 0.43, p = 0.012; see **Figure 2** for plot of uncorrected correlation and **Figure 3** for representative examples). 3 year hippocampal volume *change* analysis revealed a significant *protective* correlation with total LEQ after the addition of baseline volume as a covariate (r = 0.41, p = 0.02).

A repeated measures ANCOVA controlling for age, gender and hypertension, tested the interaction between TIME×LEQ group in hippocampal volume, and confirmed the prior result: F = 4.47 p = 0.042.

To examine whether the association with change in hippocampal volume was regionally specific or non-specific, the above analysis was repeated using Whole Brain Volume (WBV) as the dependent variable. No significant relationship was found at any time point or as a function of change (**Table 2**). Similar null results were found when whole brain WMH measures were used (**Table 2**).

Baseline Geriatric Depression Scale (GDS) scores were compared between LEQ groups to ensure that low mood was not a confounding issue. T-test found no difference in this respect (Low LEQ mean = 1.06 SD = 1.47; High LEQ mean = 1.55 SD = 2.07; t-test value = 0.75, p-value = 0.46).

Finally, in order to exclude the possibility that incipient AD was manifesting as a drop-off of *current* complex mental activities, we repeated the correlation test between hippocampal volume change and the LEQ derived from the young adulthood and middle-age life stages (whilst controlling for the same set of covariates). These partial correlations were no less robust than when the full LEQ was used (hippocampal change.young adulthood LEQ r = 0.43, p = 0.014; hippocampal change.mid-life LEQ r = 0.38, p = 0.028).

## Discussion

High LEQ individuals experienced an average loss of 3.6% of hippocampal volume over a three-year period, whilst low LEQ individuals exhibited more than twice this volumetric loss (8.3%). Furthermore, a negative correlation between LEQ and hippocampal shrinkage was noted, indicating that in general those with higher complex mental activity experienced less prospective atrophy in this region. These findings are of interest because the hippocampus is particularly susceptible to atrophy in early AD, changes which are in fact prognostic for the development of dementia in at risk individuals ([24], [25], [16], [17]. Lifespan mental activity may therefore afford specific neuroprotective advantages for hippocampal integrity, however a number of potential confounding issues first need to be considered.

The possibility that preclinical AD change was differentially affecting our high and low LEQ groups at the start of the study was addressed in two ways. Firstly, baseline volume was controlled for in all multivariate change analyses. Second, a re-analysis restricted to LEQ scores in the Young Adulthood and Mid-Life epochs found that correlations with hippocampal volume loss were fully conserved.

Given the link between stress, depression and loss of hippocampal neurons [26], the possibility that hippocampal atrophy was being mediated by mood differences in our LEQ groups was evaluated. We observed no significant differences, or appropriate trends, on the Geriatric Depression Scale in those with high or low lifetime mental activity and so this factor is not a likely explanation.

The divergent pattern of hippocampal atrophy seen in the high and low LEQ groups was not replicated in whole brain and WMH volumetric measures, intimating a degree of topographic specificity. A hippocampus-specific, activity-dependent neuroprotective mechanism has been suggested in a trial of cognitive training. Using *in vivo* proton spectroscopy, healthy older individuals who underwent 5-weeks of memory exercises benefited from increased levels of phosphocreatine-creatine in the medial temporal lobe specifically [27]. Interestingly, exogenous phosphocreatine is neuroprotective in animal models [28] and appears to assist in neuropsychological test performance [29].

Our results suggest that individuals with greater lifespan complex mental activity are at less risk for hippocampal atrophy and so provides primary *in vivo* human evidence for a structurally protective mechanism relevant to brain reserve theory. These findings are reminiscent of a MRI study of younger adults that found larger hippocampal volumes in those who repeatedly encode and retrieve spatial memories (e.g., taxi-drivers) than in matched peers [30].The alternative that high LEQ individuals ‘started off’ with larger hippocampi is less compelling because control of intracranial volume did not affect our results. Epidemiological studies [1] and clinical trials [3] that have linked increased complex mental activity to decreased dementia risk and decreased cognitive decline may therefore potentially be due to a neuroprotective effect in the medial temporal lobe.

Given the protective association between complex mental activity and hippocampal atrophy was evident using LEQ scores from all three lifestages in combination, *lifelong* participation in diverse and stimulating mental activities may be required for maximal hippocampal protection. Moreover, given the low likelihood that complex mental activities may be harmful, and the prospects that increased participation may reduce risk for age-related cognitive decline, this data adds further impetus to interventions aimed at increasing educational, occupational and cognitive lifestyle opportunities during all phases of the lifespan.

There are a several potential biological mechanisms that could underlie an activity-dependent neuroprotective effect in the hippocampus. At the molecular level, environmental enrichment has been found to upregulate neurotrophic hormones such as BDNF [31] [32], increase post synaptic glutamatergic AMPA receptors critical for long term potentiation [33], and increase expression of neprilysin, a major beta-amyloid degrading enzyme [8]. Furthermore, each of these effects have been described as more pronounced in the hippocampus (for a review of this topic see reference[5]. At the cellular level, effects of complex mentation are primarily neurorestorative, with mounting evidence for stimulation of *de novo* neurogenesis in the hippocampus [34], as well as synaptogenesis [35] and angiogenesis [36].

A limitation of this analysis was the level of attrition in the original sample, particularly in individuals consenting to repeat MRI. In order to rule out any systematic bias, we compared the follow-up groups that did or did not complete brain imaging on a number of demographic and cognitive variables and could not detect any significant differences. We had also expected to find hippocampal volume differences between high and low mental activity groups at baseline that became more pronounced three years later, yet differences were only discernible at follow-up. It is unlikely that atrophic rates begun to diverge solely during our study. What is more plausible is that the passage of time enabled subtle differences in hippocampal volume to increase to within the limits of our measurement error. Image quality, for example, improved somewhat during the course of the study, and this may account for the modest reduction in coefficient of variance for hippocampal volumes from 0.125 at baseline to 0.108 three years later.

This study examined the longitudinal course of volumetric brain changes in a group of healthy elderly individuals relative to their lifespan pattern of complex mental activity. Evidence of a link between such mental activity and diminished hippocampal atrophy was found, with those with more complex activity backgrounds exhibiting less than half the amount of atrophy than those with less activity. It is therefore theorized that complex mental activity may contribute to defense against cognitive impairment via a neuroprotective mechanism in the medial temporal lobe. These findings may be relevant to better understanding the brain reserve phenomenon observed in epidemiological and clinical studies and so further research on the topic is required.

## Materials and Methods

### Participants

Healthy control individuals participating in the Sydney Stroke Study comprised the present sample. The Sydney Stroke Study is a 5-year longitudinal examination of cognitive, medical, psychiatric and brain imaging changes. Details of the recruitment, selection and assessment process were published elsewhere [19]. Briefly, 103 unpaid healthy volunteers aged greater than 60 years from the local areas of two major teaching hospitals in Sydney were recruited from community groups during 1997–2000 and gave written informed consent. The presence of a medical history of dementia, stroke, transient ischemic attack, alcohol abuse, or other major neurological or psychiatric condition affecting cognition was exclusionary. Each individual underwent a comprehensive neuropsychological assessment, medical history and review, psychiatric and psychosocial interview as well as structural magnetic resonance imaging (MRI) at baseline. Clinicodemographic variables reported here include years of formal education, the Mini Mental State Examination (MMSE), the National Adult Reading Test (revised) estimate of premorbid intelligence (NART-IQ) and the Geriatric Depression Scale (GDS). Hypertension was assessed by self report questions concerning medical diagnosis and medication use.

Each of these evaluations was repeated between 36–40 months following the initial baseline set of assessments. 79 of the original 103 who entered the study provided a complete Lifetime of Experiences Questionnaire (LEQ) at baseline. Of these, 59 completed baseline MRI. At 3 year follow-up, 70 individuals presented for clinical evaluation and 37 agreed to and completed repeat MRI. The latter group of 37 individuals comprised the current sample of interest.

### Neuromorphologic Measures

#### MRI

was performed on a 1.5 T Signa GE scanner (GE Systems, Milwaukee, USA) using the following protocol: A scout mid-sagittal cut (2D, TR 300 msec, TE 14 msec; 5 mm thick, number of excitations 1.5); 1.5 mm thick T1-weighted contiguous coronal sections through whole brain using a FSPGR sequence and 3D acquisition (TR 14.3 msec, TE 5.4 msec); 4 mm thick (0 skip) T2-weighted FLAIR coronal slices through whole brain (TR 8900, TE 145, TI 2200, FOV 25, 256×192). MRI was conducted at baseline and at three years follow-up.

#### Intracranial & Whole Brain Volume

T1-weighted images were processed using a semi-automated segmentation procedure based on SPM2 (Wellcome Department of Cognitive Neurology, University College London, UK) to calculate intracranial volume (ICV) and whole brain soft tissue volume = gray matter+white matter volume (WBV) [20].

#### White Matter Hyperintensity (WMH) Volume

FLAIR-weighted images were processed using a semi-automated algorithm developed and published by our group [20].

#### Hippocampal Volumetry

A manual tracing protocol for identifying and measuring the hippocampus on T1-weighted images was adapted from Watson et al [21] and has been published before [22]. Images were transferred to a workstation and analysed using Analyze© (Version 4.0; Imaging Resource Center, Mayo Clinic, Rochester MN) software. Hippocampus was defined supero-anteriorly by the amygdala, laterally by the inferior horn of the lateral ventricle, medially by entorhinal cortex/choriodal fissure, and posteriorly by the slice where the crus of fornix is seen to separate from the wall of the lateral ventricle. Rater test-retest reliability was 0.995. All raters were blind to participants' clinical details and their complex mental activity status profile. Hippocampal volumes referred to throughout represent averages across right and left sides.

### Estimate of Lifespan Complex Mental Activity

Lifespan mental activity was estimated using the Lifetime of Experiences Questionnaire (LEQ). The LEQ measures complex mental activity levels in the domains of education, occupation, creative arts, reading, writing, socializing and day-to-day habits in each of the life stages of young adulthood (13–30 years of age), middle age (30–65 years) and late life (post 65 year), Each lifestage produces a subscore and these are summed to produce a total score. The LEQ is therefore a part-retrospective and part-contemporaneous measure whereby higher LEQ scores indicate a higher level of complex mental activities across the lifespan, The LEQ has been shown to be a reliable and valid measure of complex mental activity: Item Response Theory estimates of reliability ranged from 0.85–0.92, the test-retest reliability correlation coefficient was 0.98, and higher LEQ scores independently predicted decreased cognitive decline over 18 months (r = 0.37) [23]. The same sample is being reported here at 3 year follow-up with the addition of serial MRI., LEQ ranged in this sample from 29.30–130.3 (mean = 78.7, SD = 24.2).

### Analysis

The main question under test was the relationship between baseline LEQ and three-year prospective change on hippocampal volume. We hypothesized that higher LEQ at baseline would be specifically related to less hippocampal atrophy at baseline and at three year follow-up. This was anticipated to be a regionally specific finding, and so no difference was expected between LEQ groups on whole brain measures of brain volume or WMHs.

We tested these hypotheses by multiple regression analyses in the form of partial correlations, controlling for the covariates of age, gender, hypertension, baseline volume and ICV. We also planned to compare baseline and follow-up hippocampal volumes in high and low LEQ groups in a repeated measures ANCOVA (controlling for age, gender, hypertension, baseline volume and ICV). Multiple comparison correction was applied within each analysis using the SPSS package (14.0 for Windows).

## References

[1] Valenzuela MJ, Sachdev P (2006). Brain Reserve and Dementia: A Systematic Review.. Psychological Medicine.

[2] Valenzuela M, Sachdev P (2006). Brain Reserve and Cognitive Decline: A Nonparametric Systematic Review.. Psychological Medicine.

[3] Valenzuela M, Sachdev P (2008). Can cognitive exercise prevent the onset of dementia? A systematic review of randomized clinical trials with longitudinal follow up.. American Journal of Geriatric Psychiatry in press.

[4] Valenzuela MJ (2008). Brain reserve and the prevention of dementia.. Current Opinion in Psychiatry.

[5] Valenzuela M, Breakspear M, Sachdev P (2007). Complex Mental Activity and the Ageing Brain: Molecular, Cellular and Cortical Network Mechanisms.. Brain Research Reviews.

[6] Spires T, Grote H, Varshney N, Cordery P, van Dellen A (2004). Environmental enrichment rescues protein deficits in a mouse model of Huntington's disease, indicating a possible disease mechanism.. Journal of Neuroscience.

[7] Nithianantharajah J, Hannan A (2006). Enriched environments, experience-dependent plasticity and disorders of the nervous system.. Nature Reviews Neuroscience.

[8] Lazarov O, Robinson J, Tang Y, Hairston I, Korade-Mirnics Z (2005). Environmental Enrichment Reduces Aß Levels and Amyloid Deposition in Transgenic Mice.. Cell.

[9] Cracchiolo J, Mori T, Nazian S, Tan J, Potter HG (2007). Enhanced cognitive activity-over and above social or physical activity-is required to protect Alzheimer's mice against cognitive impairment, reduce Ab deposition, and increase synaptic immunostaining.. Neurobiology of Learning & Memory.

[10] Adlard P, Perreau V, Pop V, Cotman CW (2005). Voluntary exercise decreases amyloid load in a transgenic model of Alzheimer's Disease.. Journal of Neuroscience.

[11] Janowsky J, Melnikova T, Fadale D, Xu G, Slunt G (2005). Environmental enrichment mitigates cognitive deficits in a mouse model of Alzheimer's Disease.. Journal of Neuroscience.

[12] Arendash G, Garcia M, Costa D, Cracchiolo J, Wefes I (2004). Environmental enrichment improves cognition in aged Alzheimer's transgenic mice despite stable b-amyloid deposition.. Neuroreport.

[13] Snowdon D, Kemper S, Mortimer J, Greiner L, Wekstein D (1996). Linguistic Ability in Early LIfe and Cognitive Function and Alzheimer's Disease in Late Life.. JAMA.

[14] Coyle J (2003). Use it or lose it-do effortful mental activities protect against dementia?. New England Journal of Medicine.

[15] Braak H, Braak E (1991). Neuropathological stageing of Alzheimer-related changes.. Acta Neuropathologica.

[16] Jack C, Petersen R, Xu Y, O'Brien P, Smith G (1999). Prediction of AD with MRI-based hippocampal volume in mild cognitive impairment.. Neurology.

[17] Gosche K, Mortimer J, Smith C, Markesbery W, Snowdon D (2002). Hippocampal volume as an index of Alzheimer neuropathology: findings from the Nun Study.. -Neurology.

[18] Draganski B, Gaser C, Busch V, Schuierer G, Bogdahn U (2004). Neuroplasticity: changes in grey matter induced by training.. *Nature*.

[19] Sachdev P, Brodaty H, Valenzuela M, Lorentz L, Looi J (2004). The neuropsychological profile of vascular cognitive impairment in stroke and TIA patients.. Neurology.

[20] Wen W, Sachdev P (2004). The topography of white matter hyperintensities on brain MRI in healthy 60- to 64-year-old individuals.. Neuroimage.

[21] Watson C, Jack C, Cendes F (1997). Volumetric magnetic resonance imaging: clinical applications and contributions to the understanding of temporal lobe epilepsy.. Archives of Neurology.

[22] Anstey K, Maller J, Meslin C, Christensen H, Jorm A (2004). Hippocampal and amygdalar volumes in relation to handedness in adults aged 60–64.. Neuroreport.

[23] Valenzuela MJ, Sachdev P (2007). Assessment of complex mental activity across the lifespan: development of the Lifetime of Experiences Questionnaire.. Psychological Medicine.

[24] Trollor JN, Valenzuela MJ (2001). Brain ageing in the new millennium.. Australian & New Zealand Journal of Psychiatry.

[25] Raz N, Craik F, Salthouse T (2000). Aging of the Brain and its Impact on Cognitive Performance: Integration of Structural and Functional Findings.. The Handbook of Aging and Cognition.

[26] Duman R, Heninger G, Nestler E (1997). A molecular and cellular theory of depression.. American Journal of Psychiatry.

[27] Valenzuela MJ, Jones M, Wen W, Rae C, Graham S (2003). Memory training alters hippocampal neurochemistry in healthy elderly.. Neuroreport.

[28] Brustovetsky N, Brustovetsky T, Dubinsky J (2001). On the mechanisms of neuroprotection by creatine and phosphocreatine.. Journal of Neurochemistry.

[29] Rae C, Digney A, McEwan S, Bates T (2003). Oral creatine monohydrate supplementation improves brain performance: a double-blind, placebo-controlled, cross-over trial.. Proceedings of the Royal Society of London-Series B: Biological Sciences.

[30] Maguire E, Gadlan D, Johnsrude I, Good C, Ashburner J (2000). Navigation-related structural change in the hippocampi of taxi drivers.. Proceeding of the National Academy of Sciences, USA.

[31] Pham T (1999). Changes in brain nerve growth factor levels and nerve growth factor receptors in rats exposed to environmental enrichment for one year.. Neuroscience.

[32] Ickes B, Pham T, Sanders L, Albeck D, Mohammed A (2000). Long-term environmental enrichment leads to regional increases in neurotrophin levels in rat brain.. Experimental Neurology.

[33] Naka F, Narita N, Okado N, Narita M (2005). Modification of AMPA receptor properties following environmental enrichment.. Brain & Development.

[34] Kempermann G (2006). Adult Neurogenesis..

[35] Nithianantharajah J, Levis H, Murphy M (2004). Environmental enrichment results in cortical and subcortical changes in levels of synaptophysin and PSD-95 proteins.. Neurobiology of Learning & Memory.

[36] Isaacs K, Anderson B, Alcantara A, Black J, Greenough W (1992). Exercise and the brain: angiogenesis in the adult rat cerebellum after vigorous physical activity and motor skill learning.. Journal of Cerebral Blood Flow & Metabolism.

